# Bacterial interactions with platelets: defining key themes

**DOI:** 10.3389/fimmu.2025.1610289

**Published:** 2025-07-03

**Authors:** Hammodah R. Alfar, Sidney W. Whiteheart

**Affiliations:** Department of Molecular and Cellular Biochemistry, University of Kentucky, Lexington, KY, United States

**Keywords:** GPVI, FcRγIIA, GPIB, and GPIIb/IIIa, immune responses, platelet activation

## Abstract

As first responders to vascular injury and microbial invasion, platelets play a critical role in hemostasis and immunity. Previous reviews have explored how different platelet receptors can be activated by various bacterial proteins, yet strain-specific perspectives remain underexplored. In this review, we highlight eight bacterial strains that have been associated with thrombosis, each possessing unique proteins or toxins capable of activating or modulating platelets. We discuss some common themes in the molecular interactions between these bacterial components and their effects on platelet function. Some interactions influence platelet aggregation, granule secretion, pro-inflammatory cytokine release, and thrombo-inflammatory responses, while others only mediate bacterial survival. By focusing on strain-specific mechanisms, this review provides an understanding of the different strategies employed by bacteria to manipulate platelet functions. These insights may aid in developing targeted therapeutic interventions to mitigate platelet-associated complications during bacterial infections.

## Introduction

As abundant, circulating, vascular guards, platelets are uniquely positioned to detect and respond to vascular damage to stop bleeding and maintain vascular homeostasis. These same qualities make platelets valuable sensors of circulating pathogens. The platelets’ abilities to bind and potentially endocytose pathogens (depending on size), become activated, and secrete a host of bioactive molecules suggest that they can be a pivotal part of the response to an infection. Interactions between platelets and viruses or bacteria have long been known, but their significance to immune responses has only recently become the focus of research.

Bacteria are well-known pathogens that can cause diseases with thrombotic complications, *e.g.*, infective endocarditis, pneumonia, sepsis, and hemolytic uremic syndrome (HUS). They exhibit diverse abilities to interact with platelets and can induce platelet activation, adhesion, aggregation, and secretion. Some species appear to interact with platelets via multiple pathways. Previous reviews have focused on the many platelet surface receptors and how they bind pathogens. Here, we take a bacteria-centric view examining how eight different bacterial pathogens affect platelets through direct and indirect binding, secretion of bacterial proteins, and internalization. We specifically address how each species uses different mechanisms to affect platelet function and cause cardiovascular complications.

### Platelets in hemostasis and immune response

Platelets are known for their roles in hemostasis and thrombosis (1–3). Upon tissue damage, they are part of the first response to vascular injury. They prevent further blood loss and limit the invasion of pathogens into circulation. Platelets also contribute to healing the injured area and limiting infection through the release of growth factors and microbicidal peptides from their granules (4). Though lacking nuclei, platelets contain most typical cellular organelles (*e.g.*, mitochondria, endosomes, and granules), which contribute to their function in hemostasis (5, 6). Platelets have three types of granules: alpha (α), dense (δ), and lysosomal, which contain various small molecules, cytokines, chemokines, clotting factors, and enzymes, that are released upon platelet activation, are essential for function, and may contribute to pathology (4, 7–14). In the last decade, an additional secretory granule (T-granules) with tubular morphology has been proposed, which contains toll-like receptor 9 (TLR9) and protein disulfide isomerase (PDI) (15). Platelets are produced by megakaryocytes (MKs) in the bone marrow, where they are equipped with the appropriate organelles and granule contents before being released into the bloodstream (16–19). MKs in the lung have a distinct immune phenotype, but their contribution to platelet production is controversial (20–24). Recently, platelet transcriptomics data from COVID-19 and septic patients suggest that this process is altered by infection and thus may be systemically responsive to vascular health (25). Besides the proteins made by MKs, platelets also endocytose proteins from the circulation and store them in and release them from their granules (*e.g.*, fibrinogen, IgG, albumin, and fibronectin) (26, 27). Thus, platelets are able to both sample their environment via endocytosis and alter it via secretion of their granule content.

Platelets are gaining more attention for their role in immune responses to bacteria, viruses, and parasites (28–30). These roles are not surprising, as platelets express a wide range of cell surface receptors that allow them to interact with different pathogens (31, 32). After pathogen detection, platelets release cytokines, chemokines, and microbicidal peptides that kill or trap pathogens to limit their spread (33, 34). Platelets can also alert immune cells to invading pathogens through released cytokines, chemokines, and microvesicles, which enclose different molecules (*e.g.*, miRNA, RANTES/CCL5, P-selectin, defensins, kinocidins, and thymosins) (35). The microvesicles can alert immune cells, kill some pathogens, and influence gene expression in adjacent cells (*e.g.*, monocytes, smooth muscle cells, and vascular cells) (5). Additionally, surface exposure of granule membrane proteins (*i.e.*, P-selectin) drives direct interactions between platelets and circulating leukocytes (*e.g.*, neutrophils and monocytes), further integrating platelet reactivity with immune system cells. A common facet of systemic infections is reduced platelet count (mild thrombocytopenia), but the severity and underlying mechanisms vary between pathogens (36–38). Consistently, sepsis is associated with platelet consumption and decreased platelet counts, which are prognostic of poorer patient outcomes and higher risks of recurrent infections (36).

## Interactions between platelets and bacteria

The interactions between bacteria and platelets are complicated, dynamic, and evolving. Some interactions are part of the host defense system, while others affect bacterial evasion of this response. Certain bacteria directly or indirectly interact with platelets to trigger their activation or manipulate their functions, dysregulate their immune responses, or exacerbate thrombosis. While not the only mechanism by which bacteria form thrombi, bacteria can adhere to endothelial cells, disturb their permeability, and expose the procoagulant sub-endothelium, which is a normal platelet activation for hemostasis. Platelets express an array of receptors and secretory granules that enable them to recognize and respond to different bacterial species (31). Upon encountering bacteria, platelets can rapidly adhere, activate intracellular signaling pathways, and release antimicrobial substances stored within their granules to destroy the invading pathogens. The released molecules can recruit other immune cells, *e.g.*, neutrophils and monocytes, thereby contributing to the overall host defense against bacterial infections. However, bacteria-platelet complexes can shield the bacteria from antibiotics. *S. aureus* generates biofilms, which shield it from immune responses and render it more resistant to antimicrobial therapies (39). These biofilms contain proteins, polysaccharides, and extracellular DNA, which ensnare and trigger platelets, promoting their aggregation (40). This platelet activation promotes the recruitment of immune cells that secrete cytokines and tissue factors, potentially leading to organ damage if the biofilm is adjacent (39, 41). Infective endocarditis (IE) is a well-known example of a biofilm-associated disease. Its pathogenesis primarily involves the development of septic vegetations—bacterial colonies embedded within fibrin and platelet aggregates that form on heart valves (42). The presence of platelets is essential for *in vitro* biofilm formation (40). Despite these clear interactions, the significance of platelet-bacterial interaction is unclear and challenging to modulate therapeutically. Platelet responses appear essential for the immune response; however, extensive platelet activation leads to thrombotic events that exacerbate bacterial infection, enhance bacterial survival, and ultimately are detrimental to patients.

The study of platelet-bacteria interactions has a long history. In 1901, Levaditi first reported how rabbit platelets interact with *Vibrio cholera*, demonstrating that platelets aggregate when incubated with the bacteria (43). However, not until the 1970’s did Clawson and White conduct specific studies of the interactions between platelets and bacteria (44–47). More recently, platelets have been shown to endocytose bacteria such as *S. aureus in vivo* and *in vitro*, and the process is enhanced by platelet activation, but the fate of bound/endocytosed bacteria was unclear (48, 49). While this remains an active area of research, some insights were clarified with recent reports showing that platelets can kill some bacterial species (*e.g., S. aureus* and *E. coli*), but not others (*e.g.*, *S. pneumoniae)* (50, 51). Platelets kill *E. coli* in a manner enhanced by platelet factor 4 (PF4) and anti-PF4/Heparin antibodies (51), while efficient killing of *S. aureus* requires neither (50). Interestingly, platelets cannot kill *S. pneumoniae* (50). *S. pneumoniae* make platelets unresponsive to TRAP-6 stimulation and induce phosphatidylserine (PS) exposure on the platelet surface (50). The latter effect might indicate the conversion of platelets into a procoagulant form or the induction of platelet apoptosis. Such data emphasize the complexity of the interactions between platelets and bacteria, as such interactions depend on the bacterial species and strain. In addition to the pathophysiological complexity of platelets’ interaction with bacteria, experimental variations in the literature often result in contradictory data regarding the reactivity of specific bacterial strains with platelets. Some of the experimental variations are caused by the form of platelets used in experiments [*i.e.*, washed platelets or platelet-rich plasma (PRP)], platelet-to-bacteria ratio, and the platelet activation assay metric (*i.e.*, aggregation or P-selectin exposure). Hence, depending on the bacterial strain, platelets can have a positive or negative impact on bacterial spreading and survival.

### Mechanisms of platelet-bacteria interaction

While platelets appear to have several ways to interact with bacteria, there are some common themes that are used by several bacterial strains. Direct interactions between bacteria and specific platelet receptors have been demonstrated. *S. sanguinis* binds directly to GPIb (52). Other platelet receptors, such as TLRs, FcγRIIA, complement receptors, and integrins (*i.e.*, GPIIb/IIIa), can directly bind specific bacterial species (53). Platelets can bind bacteria indirectly via plasma proteins that are ligands for specific receptors (*e.g.*, von Willebrand Factor (vWF)), which bridge *S. aureus* and GPIb (54). The amount of these plasma proteins can change during pathological infections, thus altering the potential mechanisms of the interactions (55–58). Bacteria also release specific molecules (*e.g.*, toxins) that interact and affect platelets. *E. coli* and *S. pneumoniae* release Shiga toxin and pneumolysin, respectively, which are associated with platelet activation (5). Platelets can also internalize bacteria either directly or via opsonization of IgG-coated bacteria through FcγRIIA (59).

Interestingly, not all these bacterial interactions lead to platelet activation. Some play a supporting role by increasing platelet adhesion under shear conditions (60). Bacterial-induced platelet adhesion/aggregation exhibits distinctive features that differ from the responses to hemostatic and physiological agonists (60). First, unlike agonists such as ADP, bacterial-induced platelet aggregation is an all-or-none process (61). It depends on the concentration of bacteria introduced into the reaction (60). Secondly, platelets respond slowly to bacteria compared to hemostatic agonists (61). Once bacteria are introduced into the reaction, it may require 2–20 min for platelets to become activated and aggregate, in contrast to the <1 min needed when thrombin is added (61). This delay, *a.k.a.* lag time, varies based on the species, strain, and concentration of the bacteria interacting with platelets (60). Lastly, in contrast to hemostatic activation, where activation of single types of platelet receptors is sufficient, activation by bacteria often involves co-stimulation of the FcγRllA (53).

## Platelet interactions with *Staphylococcus aureus*

*S. aureus* is a spherical gram-positive bacterium, commensal on the skin and mucous surfaces. Once in the bloodstream, it is a hazardous pathogen capable of inducing necrotizing infections marked by extensive inflammation and tissue damage. This is due to its ability to secrete proteins and enzymes such as proteases, lipases, nucleases, and hyaluronidase, which degrade surrounding tissues (62). *S. aureus* is equipped with elements that protect it from the immune response generated against tissue damage and is capable of escaping the immune system in several ways, such as the release of chemotaxis inhibitors, leukocyte toxins, complement inactivators, and other antimicrobial peptides (62).

Severe *S. aureus* infections have been linked to a higher risk of thrombosis, especially deep vein thrombosis (DVT) and disseminated intravascular coagulation (DIC), as the bacteria can have effects on the pro-coagulant and inflammatory pathways and on the anticoagulation factors (63, 64). *S. aureus* was the first bacterium shown to be endocytosed by platelets, but ADP was required (5, 44, 49). Platelets bind and extend their pseudopods to encapsulate *S. aureus*, thus limiting bacterial dissemination into the bloodstream (65). Platelets are involved in the eradication of systemic infections (65). Under normal conditions, platelets patrol Kupffer cells (KCs) through a “touch and go” mechanism that involves GPIb and vWF at the KC surface (66). This process is intensified during infection, where platelets are the first cells to arrive in an infected liver, even before neutrophils (66). KCs capture *S. aureus* and platelets switch from “touch and go” mechanism to “sustained GPIIb/IIIa-dependent adhesion”, which traps the bacteria and limits their spread (66). This process limits liver dysfunction and is essential for *S. aureus* eradication and host survival (66). Platelets are also involved in the generation of a more specific immune response in a process that depends on GPIb and C3. This directs some bacteria to the spleen to activate CD8α^+^ dendritic cells (67). *S. aureus* can also induce platelet aggregation and apoptosis. The aggregation response is unique compared to other bacterial species. *S. aureus* induces aggregation with a shorter lag time (2–5 min) than other species, such as *S. sanguinis* or *S. gordonii* (15–20 min) (68, 69). Apoptosis induction is mediated through the degradation of Bcl-xL protein (anti-apoptosis protein), which increases the exposure of PS, which supports the coagulation system (36).

**Figure 1:**
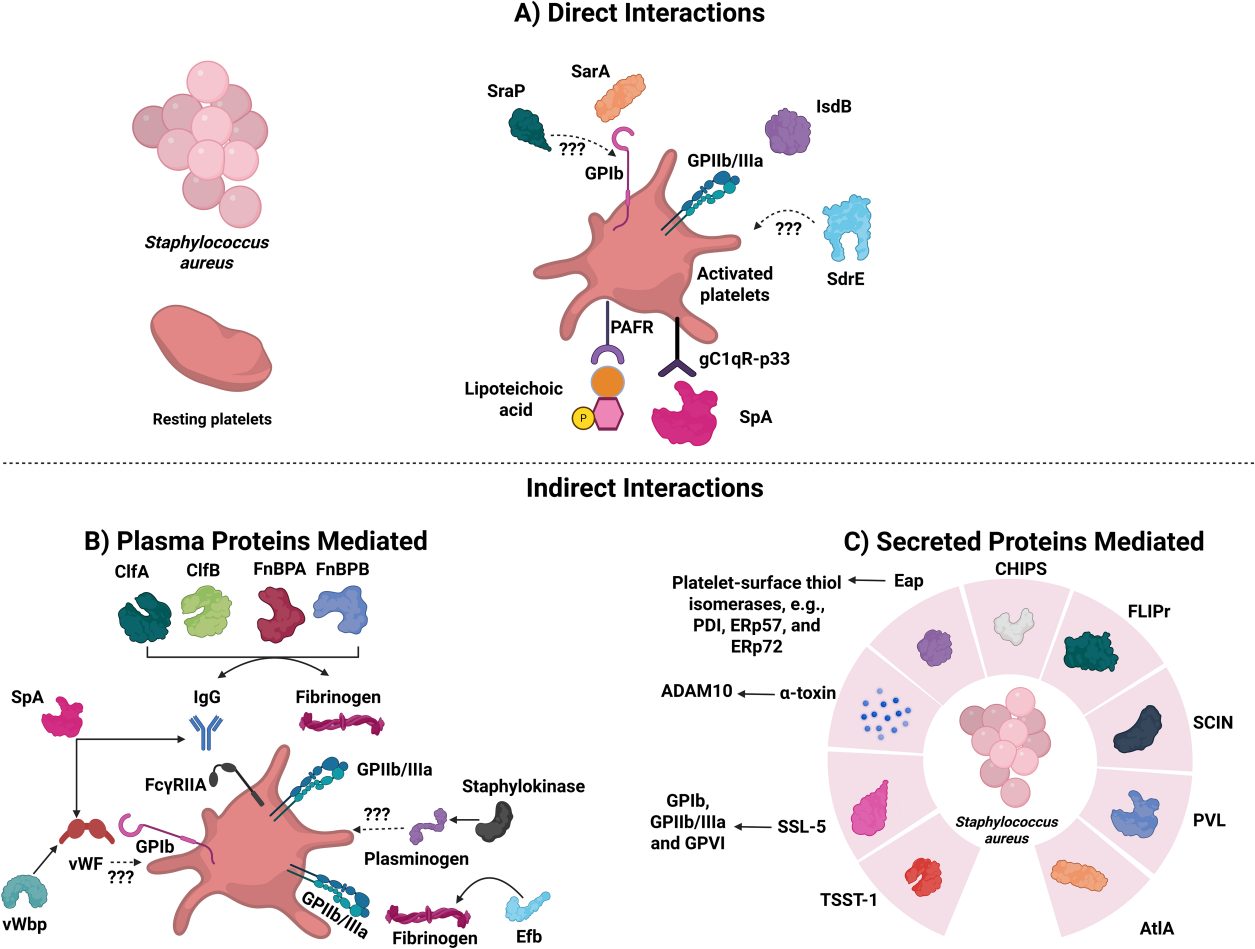
*Staphylococcus aureus* proteins that are involved in direct or indirect interactions with platelets. The diagram depicts known platelet-*S. aureus* direct and indirect interactors. **(A)** *S. aureus* proteins capable of activating directly. SraP, Serine-rich adhesin protein; SarA, Staphylococcal accessory regulator protein; IsdB, Iron-responsive surface determinant B; SdrE, Serine-aspartate repeat protein; SpA, Staphylococcal protein A; PAFR, Platelet-activating factor receptor. **(B)** *S. aureus* proteins capable of activating platelets indirectly (via plasma proteins). Abbreviations are: ClfA and ClfB: Clumping factors A and B; FnBPA and FnBPB: Fibronectin-binding proteins A and B; SpA: Staphylococcal protein A; vWF: von Willebrand Factor; vWbp: vWF-binding protein; and Efb: Extracellular fibrinogen binding protein. **(C)** *S. aureus* proteins capable of activating platelets indirectly (via secreted proteins). TSST-1, Toxic shock syndrome toxin-1; SSL-5, Superantigen-like-5; ADAM10, Disintegrin and metalloproteinase domain-containing protein 10; PDI, Protein disulfide isomerase; Eap, Extracellular adherence protein; CHIPS, Chemotaxis inhibitory protein of *S. aureus*; FLIPr, Formyl peptide receptor-like 1 inhibitory protein; SCIN, Staphylococcal complement inhibitor; PVL, Panton-valentine leucocidin; and AtlA, Major autolysin.

*S. aureus* modulates thrombosis through a diverse array of surface elements, which in most cases fall into two categories: Microbial Surface Components Recognizing Adhesive Matrix Molecules (MSCRAMMs) and Secretable Expanded Repertoire Adhesive Molecules (SERAMs). MSCRAMMs are connected to the peptidoglycan through a sortase-anchoring motif, while SERAMs are affixed to the bacterial cell surface through non-covalent means (70). Using these proteins, *S. aureus* modulates thrombosis (induction or resolution) through the mechanisms discussed below (see **Figure 1**).

### Direct platelet interactions

Platelets express receptors that directly bind *S. aureus* without an adapter and induce activation. Through GPIb and GPIIb/IIIa, platelets bind to the staphylococcal accessory regulator protein (SarA) and Iron-responsive surface determinant B (IsdB), respectively (39, 71). The presence of both of these proteins is essential for bacterial adherence to platelets and aggregation (71, 72). Platelets also express the complement receptor, gC1qR-p33, which, besides binding to a plethora of plasma proteins, binds to the staphylococcal protein A (SpA) (73, 74). Under resting conditions, platelets express minimal gC1qR levels, but this increases substantially upon activation with TRAP, epinephrine, or ADP (75). This receptor plays a significant role in the pathogenesis of IE, which can be caused by *S. aureus* (76). Another protein that can mediate binding and activation of platelets directly is the highly glycosylated serine-rich repeat (SRR) protein called Serine-rich adhesin Protein (SraP) (73). The presence of the SRR on *S. aureus* suggests it could interact with GPIb, but this has not been confirmed (53, 73). Other substances released by platelets, like ADP, thromboxane A_2_, and PF4, can increase the impact that bacteria have on platelet activation (77). Arman et al. showed that released PF4, upon interaction with *S. aureus*, is essential for platelet aggregation and reduces its lag time (77). Finally, Serine-aspartate repeat protein (SdrE), associated with *S. aureus* cell walls, also binds platelets and can induce their aggregation (78).

### Indirect platelet interactions involving plasma proteins

*S. aureus* binds to various extracellular matrix proteins—such as fibrinogen, fibronectin, vWF, laminin, vitronectin, complement proteins, collagen, IgG, and thrombospondin—which can act as bridges, allowing platelets to interact with the bacteria (72, 79–81). SpA, which binds directly to gC1qR-p33, binds both vWF and the Fc region of IgG and can activate both GPIb and FcγRllA, respectively (79–81). Molecules such as Clumping factors A and B (ClfA and ClfB) and fibronectin-binding proteins A and B (FnBPA and FnBPB) bind to fibrinogen and act as bridging molecules (82). These proteins are expressed and produced during various stages of the bacterial growth cycle, bind to different fibrinogen chains, and induce platelet activation and aggregation (82). ClfA binds to the C-terminus of the fibrinogen γ chain, while ClfB binds to the C-terminus of the fibrinogen α chain (82). FnBPA and FnBPB bind to the C-terminus of the γ chain of fibrinogen (73, 82). Plasma IgG can bind to ClfA, ClfB, FnBPA, and FnBPB on *S. aureus*, leading to platelet activation through FcγRIIA, while complement proteins provide an additional pathway for ClfA- and ClfB-mediated platelet activation, though the specific receptor involved remains unknown (73, 83).

### Indirect platelet interactions: via proteins and α-toxin secretion

*S. aureus* secretes different toxins that can cause organ failure (*e.g.*, leukotoxin ED, superantigens, and α-type phenol-soluble modulins (PSM)) (84), though none directly interacts with platelets (84). *S. aureus* secrete a small β-barrel pore-forming toxin known as α-toxin (Hla; *a.k.a.* α-hemolysin due to its ability to lyse red blood cells) that can activate platelets (85). α-Toxin is initially secreted in a water-soluble monomeric form, but once bound to a membrane, it oligomerizes to a heptamer with a diameter between 1–3 nm (5, 86). The formed pore allows the influx of Ca²^+^, K^+^, ATP, and smaller molecules (between 1 and 4 kDa) (87). In 1964, Siegel and Cohen showed that α-toxin induces the aggregation of human platelets and a procoagulant response when present at sub-lytic concentrations (88). Recent reports have shown that, in addition to platelet aggregation and activation, α-toxin induces platelet apoptosis, platelet-neutrophil aggregate formation, aggregated platelet deposition in the liver, and initiates platelet protein synthesis (84, 89–92).

α-Toxin binds to ADAM10 on platelet surfaces to form an active, zinc-containing, metalloprotease complex (84). Though α-toxin activates platelets, it eventually destroys them, mediating lysis and impaired thrombus stability (93). Active ADAM10 cleaves the extracellular domain of GPVI, triggers platelet secretion, and impairs the subsequent events of platelet activation, such as platelet aggregation and adhesion to fibrinogen and vWF (94, 95). The interaction between ADAM10 and α-toxin precipitates acute lung injury and hemorrhage in mice, through disruption of GPIIb/IIIa activation, also mediated by GPVI proteolysis (94). As a response to α-toxin, human platelets release β-defensin 1, a granule cargo protein that impairs the growth of *S. aureus* and induces neutrophil extracellular traps (NETs) formation. NET formation limits *S. aureus* growth (65). However, they can be degraded by α-toxin (96). *S. aureus* expresses many virulent factors that thwart the microbicidal activity of NETs (97, 98).

Another toxin released from *S. aureus* is toxic shock syndrome toxin-1 (TSST-1), which causes thrombocytopenia, platelet activation, and apoptosis *in vivo*, though the effects on isolated platelets are limited *in vitro* (99). Certain strains of *S. aureus* are positive for the panton-valentine leukocidin (PVL) toxin, which damages neutrophils and leads to platelet activation via neutrophil release of α-defensins and the myeloperoxidase product, hypochlorous acid (HOCl), and some HOCl-modified proteins (100). While some of these toxins directly affect platelets, it is unclear whether the damage they cause to other cells also precipitates platelet activation through the production of damage-associated molecular patterns (DAMPs).

*S. aureus* also activates platelets through the secretion of other proteins (39). Extracellular adherence protein (Eap; a SERAM (101)) binds to the platelet-surface thiol isomerases (*e.g.*, PDI, ERp57, and ERp72), and promotes activation and aggregation (102). Eap also induces the binding of plasma proteins such as fibrinogen, TSP-1, vitronectin, and fibronectin in a time, concentration, and temperature-dependent manner (102). *S. aureus* secretes chemotaxis inhibitory protein of *S. aureus* (CHIPS), formyl peptide receptor-like 1 inhibitory protein (FLIPr), staphylococcal complement inhibitor (SCIN), and the major autolysin (AtlA) proteins, which all promote platelet activation and aggregation (70, 103). Superantigen-like-5 (SSL-5) is released by *S. aureus* and induces platelet aggregation through GPIb and GPIIb/IIIa, as well as increases the platelet adhesion to the endothelial cell matrix (103). This activation is attributed to SSL-5 binding to GPVI (104). However, SSL-5 can bind P-selectin glycoprotein ligand-1 (PSGL-1) to inhibit neutrophil rolling and migration to infected sites (105).

### Direct activation of the coagulation system

*S. aureus* can directly trigger the coagulation cascades through its two prothrombin activators: staphylocoagulase and vWF-Binding Protein (vWbp) (106). Both activate prothrombin, leading to the formation of active staphylothrombin, which produces fibrin and can protect *S. aureus* against the host’s defense mechanisms (106). vWbp plays a role in the vascular adhesion of *S. aureus* through two distinct mechanisms: first, by binding to vWF under shear conditions, and second, by activating prothrombin, resulting in the formation of *S. aureus*-fibrin-platelet aggregates through the interaction with GPIIb/IIIa (79).

### *S. aureus* can prevent thrombosis

*S. aureus* contains a staphylokinase that promotes the dissolution of blood clots (97). Staphylokinase binds to plasminogen with high affinity and converts the zymogen into plasmin, which cleaves fibrin (106). Additionally, *S. aureus* secretes an extracellular fibrinogen binding protein (Efb), which binds to fibrinogen via its N-terminus, to C3, through its C-terminus, or directly to P‐selectin on activated platelets (107, 108). These three interactions can lead to different outcomes. The binding of the N-terminus of Efb to P-selectin inhibits platelet interactions with PSGL‐1 on monocytes and neutrophils and their recruitment (107, 108). The inhibitory effect of Efb on platelet function is harmful to the host, as platelet activation is essential to eradicate *S. aureus* (109). However, the binding of Efb to fibrinogen and C3 is essential for bacterial survival. This enables *S. aureus* to shield itself from phagocytosis (107, 110). Finally, the cell wall component, lipoteichoic acid from *S. aureus*, inhibits platelet aggregation in response to physiological agonists and reduces thrombosis *in vitro* by binding to platelet-activating factor receptor (PAFR) (111, 112). Interestingly, anti-TLR2 antibodies had no effect (111).

## Platelet interactions with *Streptococcus pneumoniae*

*S. pneumoniae* is a lancet-shaped, gram-positive bacterium that is a leading cause of life-threatening, community-acquired pneumonia (CAP) (5). The severity of CAP correlates with the development of thrombocytopenia (113). Besides CAP, *S. pneumoniae* can cause sepsis and, on rare occasions, IE (114–116). Recent studies have shown that a significant portion of CAP-associated fatalities may be linked to cardiovascular incidents occurring during infection (117, 118). Such events have various causes, including *S. pneumoniae* itself and its virulence factors, but there is a growing recognition that platelet activation contributes (119).

In the 1970s, the interaction between platelets and *S. pneumoniae* was first suggested, but this has not been without controversy (46, 120). Some reports showed platelet activation and aggregation upon the addition of *S. pneumoniae*, and other reports did not (46, 120). The interaction between platelets and *S. pneumoniae* is serotype-specific, as some serotypes induced platelet activation, while others did not (46, 120). In contrast to *S. aureus*, which is killed by platelets and their releasate, *S. pneumoniae* is not killed by platelets nor their releasates, but it affects the viability of platelets as they become unresponsive to TRAP-6 stimulation and expose PS on their surfaces (50). The latter effect might indicate the conversion into procoagulant platelets or that *S. pneumoniae* induces platelet apoptosis.

**Figure 2:**
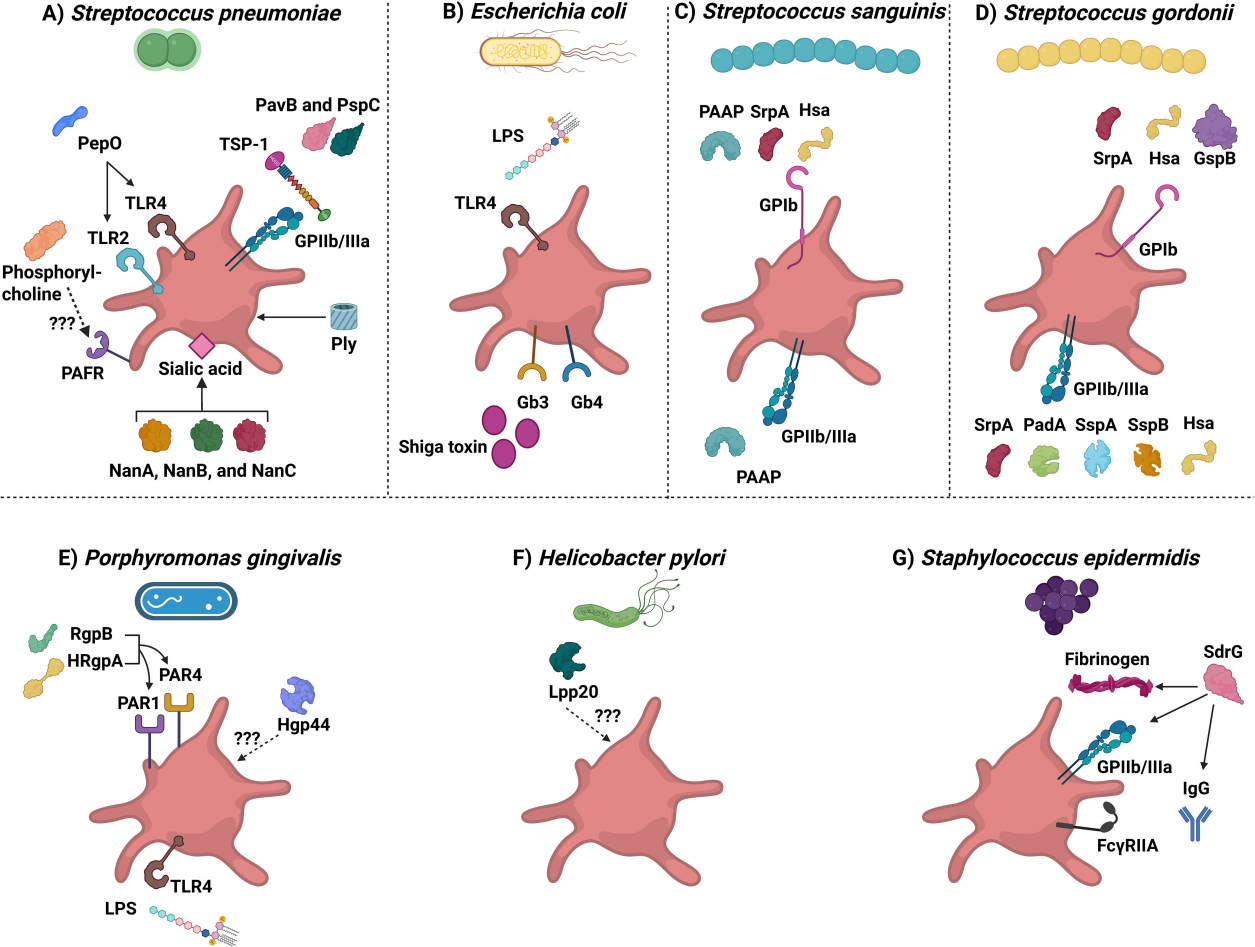
Bacterial strains derived molecules that interact with platelets. The diagram depicts different bacterial strains that can interact with platelets. **(A)** *Streptococcus pneumoniae.* Abbreviations are: PepO, pneumoniae endopeptidase O; TLR2 and TLR4, Toll-like receptor 2 and 4; PAFR, Platelet-activating factor receptor; NanA, NanB, and NanC, Neuraminidases A, B, and C; Ply, Pneumolysin; PavB, Pneumococcal adherence and virulence factor B; PspC, Pneumococcal surface protein C; and TSP-1: Thrombospondin-1. **(B)** *Escherichia coli.* Abbreviations are: LPS: Lipopolysaccharide; TLR4: Toll-like receptor 4; and Gb3 and 4: Globotriaosylceramide 3 and 4 receptors. **(C)** *Streptococcus sanguinis.* Abbreviations are: PAAP: Platelet-associated aggregation protein; SrpA: Serine-rich protein A; and Hsa: hemagglutinin salivary antigen. **(D)** *Streptococcus gordonii.* SrpA, Serine-rich protein A; Hsa, hemagglutinin salivary antigen; GspB, Gordonii surface proteins glycosylated streptococcal protein B; PadA, Platelet adherence protein A; SspA and SspB, Streptococcal surface protein A and B). **(E)** *Porphyromonas gingivalis.* RgpB, Arginine-specific protease B; HRgpA, high-molecular-weight arginine-specific gingipain **(A)** Hgp44, Hemagglutinin/adhesion domain of the Arg-gingipain A protein; PAR1 and PAR4, Protease-activated receptor 1 and 4; LPS, Lipopolysaccharide; and TLR4, Toll-like receptor 4. **(F)** *Helicobacter pylori*. Lpp20, Low molecular weight antigen. **(G)** *Staphylococcus epidermidis.* SdrG, Serine aspartate dipeptide repeat **(G)**.

Schrottmaier et al. recently showed that the phosphatidylinositol 3-kinase catalytic subunit (p110β) in platelets is essential for the innate immune responses against *S. pneumoniae* (121). The presence of p110β in platelets is essential for neutrophil activation and to prevent *S. pneumoniae* propagation (121). They also found that the inhibition or genetic deletion of p110β impairs the recruitment and phagocytosis of neutrophils and monocytes, hinders their infiltration, and enhances bacterial dissemination (121). Verschoor et al. showed that platelets can bind nonencapsulated *S. pneumoniae* in a GPIb- and C3-dependent process (67). However, platelets did not bind or direct capsulated *S. pneumoniae* to the spleen to activate CD8α^+^ T-cells (67). The presence of the capsule prevents the deposition of complement proteins on *S. pneumoniae* (67). How *S. pneumoniae* induces platelet activation is elusive, though several mechanisms are suggested. **Figure 2A** summarizes how *S. pneumoniae* interacts/activates platelets and induces prothrombotic/pro-inflammatory conditions through one or a combination of the following mechanisms.

### Pneumolysin directly mediates the activation of platelets

One virulence factor involved in platelet activation is pneumolysin (Ply). This cholesterol-dependent β-barrel cytolysin binds to target cells, assembles into the membrane, and forms pores (122). Ply plays an essential role in CAP by fostering *S. pneumoniae* colonization and invasion of the upper and lower respiratory tract (123). The effect of Ply on platelets has been controversial. Some reports indicate Ply induces platelet activation, as measured by flow cytometry (P-selectin expression on the surface of activated platelets) and aggregometry (platelet aggregation) (5). Other studies indicate that Ply induces platelet destruction, rendering them unresponsive to stimulation (124). Jahn et al. hypothesized that these controversial results might be due to the assays used (5). If Ply forms pores in platelets, more anti-P-selectin antibodies could get into the granules, making it appear that there was more α-granule exocytosis. Additionally, if the platelets are perforated, more light would pass through them during the aggregometry experiments, incorrectly suggesting an increase in aggregation (5). Thus, both types of assays could yield falsely positive results because the platelets were permeabilized (5, 124). This hypothesis is supported by scanning EM images that show perforated platelets after the addition of Ply (5, 124). Besides its activation, aggregation, and destruction effects on platelets, Ply induces the release of extracellular vesicles from platelets and causes neutrophils to secrete platelet-activating factor (PAF) and thromboxane A_2_; both are platelet activators (124–128). However, platelet aggregation is not solely due to Ply, as Ply-deficient strains of *S. pneumoniae* induce platelet aggregation similar to intact strains (129).

### Activation of platelet-activating factor receptor on the platelet surface

PAFR is believed to contribute to the binding of *S. pneumoniae* to endothelial cells (130). PAFR is present on the surface of many cells (*e.g.*, platelets, neutrophils, macrophages, and lymphocytes) and is considered to mediate inflammatory signals (131). The biological ligand of PAFR is PAF, which is released from cells such as neutrophils, macrophages, and endothelial cells (132). Phosphorylcholine, in bacterial membranes, mimics PAF and binds specifically to PAFR (133). Human platelets appear to have two binding sites for PAF (134). Nevertheless, the interactions between the platelet’s PAFR and *S. pneumoniae* are largely unexplored, and more work is needed to identify the effects of such interaction on the pathology associated with *S. pneumoniae*.

### TLR2 and TLR4 interactions

TLRs are pattern recognition receptors that recognize molecules with pathogen-associated molecular patterns (PAMPs). Platelets express TLRs on their surface (*e.g.*, TLR1, TLR2, TLR4, and TLR6) and in their endosomes (*e.g.*, TLR7 and TLR9) (135). The most studied are TLR2 and TLR4, as they are the most abundant TLRs on the platelet surface (136). While TLR2 binds lipoteichoic acids and peptidoglycan from gram-positive bacteria, TLR4 binds lipopolysaccharide (LPS) from gram-negative bacteria (137). Earlier reports showed that encapsulated *S. pneumoniae* induces platelet activation and aggregation through TLR2, but unencapsulated *S. pneumoniae* did not (5). Other reports suggested some encapsulated *S. pneumoniae* failed to induce platelet activation, while some unencapsulated strains did activate platelets (5). Keane et al. showed that TLR2, but not TLR4, is essential for *S. pneumoniae* induction of platelet aggregation by using blocking anti-TLR2 and TLR4 monoclonal antibodies, but again this conclusion was challenged as platelet activation was still observed in the presence of TLR blocking antibodies or in single or dual platelet TLR KO mice (TLR2^-/-^, TLR4^-/-^, TLR9^-/-^or TLR2,4^-/-^) and in MyD88^-/-^ mice (129, 138). Zhang et al. showed that recombinant *S. pneumoniae* endopeptidase O (PepO), induces an innate immune response in mice that is dependent on both TLR2 and TLR4 (139). Thus, the interactions between TLR2 and TLR4 on platelets and *S. pneumoniae* and their importance are still controversial.

### FcγRIIA activation

Platelets express another receptor that can be activated by *S. pneumoniae*, FcγRllA. In 2014, Arman et al. showed that platelets are activated by a range of bacteria, including *S. pneumoniae*, and showed that FcγRllA activation is needed (77). The activation depends on IgG and GPIIb/IIIa involvement and can be potentiated by the released PF4, ADP, and thromboxane A_2_ from platelets (77). To activate FcγRllA, *S. pneumoniae* must be opsonized with IgG, and the activation of FcγRllA provides a co-stimulatory signal used by *S. pneumoniae* and other pathogens (53). However, which component of *S. pneumoniae* is bound by the opsonizing IgG is undetermined.

### GPIIb/IIIa binding and activation

Recent reports indicate a direct binding of *S. pneumoniae* to platelets via soluble fibrin and thrombospondin-1 (TSP-1) secreted from activated platelets (140). It is suggested that the adherence and virulence factor B (PavB) and surface protein C (PspC) may potentially attach to platelet GPIIb/IIIa in the presence of TSP-1 on activated platelets (141, 142).

### Neuraminidases mediate complement activation and blood hemolysis

*S. pneumoniae* expresses neuraminidases (*e.g.*, NanA, NanB, and NanC) that can remove platelet surface sialic acids (143). These terminal sugars play a crucial role in factor H-mediated complement regulation on both cells and platelets, and their removal can result in uncontrolled complement activation, platelet aggregation, and destruction of red blood cells (143). Thus, *S. pneumoniae* could activate the complement cascade, induce platelet aggregation, and cause blood hemolysis through these enzymes (143).

### Other mechanisms

Other mechanisms have been proposed for *S. pneumoniae*-mediated activation of platelets. The phage-derived proteins, platelet-binding locus A and platelet-binding locus B (pblA and pblB), interact with platelet membrane gangliosides (119, 144, 145). Hydrogen peroxide, generated by the pneumococcal pyruvate oxidase, has been reported to affect platelet function (119, 146, 147). Finally, endothelial cell dysfunction resulting from *S. pneumoniae* infection and the production of vasoactive molecules like thromboxane A_2_ could also activate circulating platelets (148).

## Platelet interactions with *Escherichia coli*

The interactions between gram-negative bacteria and platelets are less studied (59). *E. coli* is a rod-shaped, gram-negative bacterium first identified by Theodor Escherich in 1885 (149). Most strains are human or animal commensals localized in the gastrointestinal tract (149). However, some have acquired virulent factors, which associate them with several human diseases such as sepsis and HUS (149, 150). HUS presents as a triad of microangiopathic hemolytic anemia, thrombocytopenia, and acute renal failure (137). *E. coli* can be divided into two main categories: intestinal and extraintestinal pathogenic *E. coli* (59). Each group has several strains, with enterohemorrhagic *E. coli* (EHEC) being the most studied strain (59). Consistent with the controversial results when studying bacteria and platelets, the interaction between *E. coli* and platelets has been extensively debated, specifically on the involvement of platelet TLR4 and FcγRIIA receptors and the effects of LPS on platelets (150–153). The effects are strain-dependent, which was confirmed as platelets or their relesates promote or inhibit the growth of different *E. coli* strains (154, 155).

Platelets can endocytose *E. coli*, pre-opsonized with IgG, through the FcγRII receptor to kill them (5). *E. coli* activates platelets through GPIIb/IIIa (150, 152) and that activation is enhanced in the presence of complement, thromboxane A_2_, and ADP (150, 152). The released PF4 by activated platelets binds to polyanions on *E. coli* to form a complex (51), which helps opsonize PF4-coated *E. coli* and mediate their killing in a GPIIb/IIIa- and FcγRIIa-dependent process (51). These interactions are more complicated, as the interactions between platelets and *E. coli* vary between individuals (150). The most common molecules that *E. coli* uses to affect platelet functions are LPS and Shiga toxin (**Figure 2B**).

### Lipopolysaccharide

Gram-negative *E. coli* has an outer membrane containing LPS, which consists of amphipathic glycoconjugates composed of a hydrophobic lipid domain linked to a central oligosaccharide and an outer polysaccharide. In macrophages, LPS binds to TLR4 in the presence of LPS-binding protein (LBP), CD14, and MD-2 (present on the extracellular domain of TLR4) (156). Platelets express TLR4 (5) and they also express other LPS signaling complex components such as MD2 and MyD88, but not CD14 (157). In children infected with EHEC, LPS is found on the surface of platelets only in children with HUS or before developing HUS; however, it is not found in children who did not develop HUS (153). This indicates platelet activation by LPS may precede HUS development.

In 2005, Andonegui et al. showed, for the first time, that LPS injections of mice induce thrombocytopenia in a TLR4-dependent manner (158). LPS stimulation of TLR4 is essential for TNF-α production, as platelet-depleted mice failed to secrete TNFα after LPS injection (159). This effect was reversed after platelet transfusion (159). Later, it was discovered that LPS induces TLR4-dependent platelet aggregation, α-granule secretion, and dense granule secretion (157). The lipid A fragment of LPS interacts with TLR4 to initiate a pro-inflammatory condition (154). LPS can also modify the protein synthesis in platelets, triggering a pro-inflammatory response through IL-1β splicing, translation, and secretion after caspase-1 processing (160, 161). Released IL-1β was only detected in microparticles (161); but it can amplify the pro-inflammatory condition that can lead to endothelial activation and tissue damage (59, 161). Pires et al. has shown that LPS enhances human platelet activation via a TLR4–PI3K–Akt–ERK1/2–PLA2 signaling pathway (162). Interestingly, platelets possess the ability to recognize and respond to distinct LPS structures, readily differentiating those from *E. coli* and *Salmonella minnesota* (163). Specifically, platelet releasate generated in response to *E. coli* LPS induces a unique cytokine secretion profile in peripheral blood mononuclear cells (PBMCs) that differs from the response elicited by *Salmonella minnesota* LPS (163). This suggests that platelets can detect danger signals via a single receptor, TLR4, and tailor their responses to differentially modulate immune reactions depending on the specific LPS structure encountered. Recently, Burkard et al. showed direct, *in vivo* evidence that GPVI is a central mediator of LPS-induced pulmonary thrombo-inflammation—promoting PNC formation, neutrophil recruitment, and NETosis—while its inhibition protects mice from LPS-induced acute lung injury and respiratory failure (164).There has been extensive debate about the *in vitro* effects of LPS on platelets—specifically, whether it activates them, primes them, or has no effect (150–153, 160, 162, 165–167). The differences might be due to the strain of *E. coli*, LPS type (smooth *vs.* rough LPS), concentration of LPS, or technical issues such as the ratio of platelets to bacteria, washed platelets *vs.* PRP, incubation times, and the assay being used, aggregation or P-selectin exposure. Several reports indicate that LPS isolated from *E. coli* O157 was the most potent using a TLR4-dependent process to modulate the secretion of stored cytokines by human platelets (153, 168). Yet, Moriarty et al. demonstrated that LPS from *E. coli* O157 does not induce platelet aggregation; however, viable *E. coli* O157 did (150). Arbesu et al. showed that *E. coli* (O18:K1) activates platelets independent of TLR4, GPIIb/IIIa, or plasma proteins (169). It should be noted that other reports suggest that LPS does not activate platelets but primes platelets to respond to lower levels of classical agonists (*e.g.*, thrombin, epinephrine, ADP, or arachidonic acid) (157, 160, 165, 166). However, these controversial results may be attributed to the presence of residual amounts of plasma CD14 in washed platelets. Platelets do not express CD14, but since soluble CD14 (sCD14) is in plasma, the presence of low quantities of plasma or serum could lead to a greater effect of LPS on platelets (170). The requirement of sCD14 might be consistent with the *in vivo* data regarding the effect of LPS on platelet activation (160, 170, 171). Still, other studies indicate that LPS inhibits platelet aggregation and decreases platelet adhesion to fibrinogen (172, 173).

### Shiga toxin

Discovered in 1897 by Kiyoshi Shiga, the strain of *E. coli*, called Shiga toxin-producing *E. coli* (STEC), causes vascular endothelial dysfunction by releasing Shiga toxin (59). In 1977, another toxin, verotoxin was discovered and so named because it killed Vero cells in culture (174). Both Shiga toxin and verotoxin are a group of cytotoxic proteins secreted from enteric pathogens that share structures and functions (174). Shiga toxin produced by the enterohemorrhagic *E. coli* O157:H7 (*E. coli* expresses somatic (O) antigen 157 and flagella (H) antigen 7) can cause HUS, which is the most common cause of renal failure in children ≤ 3 years (175). Thrombocytopenia might result from the effects of Shiga toxin on platelets as it induces platelet aggregation (176). Shiga toxin induces microthrombi formation in the kidney’s capillaries (specifically, in the glomerular capillaries) and decreases prostacyclin production by the damaged endothelial cells, which promotes platelet aggregation (175). The formed thrombi in the renal vessels significantly affect the efficacy of glomerular filtration, leading to renal failure (175). During HUS, platelets are activated, release their granule content, and are consumed via microthrombosis (59). As such, the diagnosis of HUS can be confused with disseminated intravascular coagulopathy (DIC). The main differences between the two conditions are the prothrombin time (PT), which is within the normal range or slightly extended, and fibrinogen levels, which are also normal or elevated in HUS (177).

Shiga toxin binds to glycosphingolipid receptors on the platelet surface [Globotriaosylceramide 3 and 4 receptors (Gb3 and Gb4)] (178). The interaction of Shiga toxin with platelets has been controversial, as some reports indicate that platelets bind and internalize Shiga toxin, which leads to aggregate formation, activation, morphological changes, and increased fibrinogen binding, while others failed to confirm the interactions (176, 179–184). Using different anticoagulants and methods of platelet isolation and purification, Gosh et al. later showed that the binding of Shiga toxin occurs on the surface of activated platelets but not on resting platelets (178). The method of isolating platelets was key, as the effects of harsher isolation conditions led to their activation and subsequent binding of Shiga toxin (178).

## Platelet interactions with *Streptococcus sanguinis*

*S. sanguinis* is an opportunistic bacterium that inhabits the human mouth (69). *S. sanguinis* is the most frequent causative microorganism of IE (185). Upon bloodstream entrance, *S. sanguinis* can cause several complications, such as adhering to host extracellular matrix protein and/or platelets, colonizing the heart valves and ultimately leading to IE (185, 186). *S. sanguinis* strains are divided into three categories based on their ability to induce platelet activation *ex vivo* (187, 188). Type 1: adhere and activate platelets with a short delay time, type 2: do not adhere but activate platelets with a longer delay time; and type 3: do not adhere or activate platelets (187).

The first streptococcal surface protein to bind and activate platelets to be identified was the platelet-associated aggregation protein (PAAP) (61, 189, 190). PAAP has a collagen-like epitope that can activate platelets through an uncharacterized receptor, but it is suggested to be GPIIb/IIIa or GPIb and not GPVI (61, 189–191). The interaction between platelets and *S. sanguinis* is shear-dependent and seems to be mediated through GPIb (60). Platelets isolated from Bernard Soulier syndrome patients (lacking GPIb on their platelets) failed to respond to *S. sanguinis*, and blocking antibodies against GPIb inhibited both aggregation and adhesion induced by *S. sanguinis* (187).

In addition to GPIb, platelet aggregation in response to *S. sanguinis* relies on GPIIb/IIIa and thromboxane A_2_. However, aggregation does not occur through direct binding to GPIIb/IIIa, as blocking this receptor with antagonists had no effect (187). Aggregation induced by *S. sanguinis* is mediated through both GPIIb/IIIa and GPIb, which can occur through either a vWF-independent mechanism or via glycosylated adhesions containing SRRs, such as serine-rich protein A (SrpA) and hemagglutinin salivary antigen (Hsa). Both bind to GPIb in a sialic acid-dependent manner (52, 192). The interaction with GPIb is through SrpA, which is not the only mechanism to activate platelets, as its deletion did not inhibit platelet activation but prolonged the lag time for platelet aggregation (61). In addition to GPIb, *S. sanguinis* can activate platelets in a complement-dependent process and through FcγRllA as well (193–195). However, certain strains of *S. sanguinis* stimulate the release of RANTES, PF4, sCD40L, sCD62p, and PDGF-AB from platelets, and other strains do not (196). Thus, it appears that different strains of *S. sanguinis* can induce platelet activation via different mechanisms, and they further differ in their requirements for thromboxane A_2_ or ADP for platelet activation (196). **Figure 2C** summarizes the main *S. sanguinis* proteins that can activate platelets.

## Platelet interactions with *Streptococcus gordonii*

*S. gordonii* is a commensal, oral bacterium that causes several complications (*i.e.*, IE) (197). As with other bacteria, the platelet-*S. gordonii* interaction is strain-specific (61). Some strains have a long lag time in aggregometry experiments, while others have shorter ones or fail to activate platelets altogether (61). *S. gordonii* possesses SRR adhesin proteins (*e.g.*, gordonii surface proteins glycosylated streptococcal protein B (GspB), Hsa, and SrpA), which bind to a variety of sialylated glycoproteins or the extracellular sialoglycans on GPIbα (198, 199). These SRR adhesins trigger platelet activation by interacting with platelet GPIb in a shear-dependent process (60, 198). While Hsa binds to N-linked sialic acids on GPIb and GPIIb/IIIa, GspB binds to O-linked sialic acids on GPIb on the membrane-proximal mucin-like core of GPIb (200). *S. gordonii* also induces platelet activation through the platelet adherence protein A (PadA), which specifically interacts with GPIIb/IIIa to induce platelet aggregation and adhesion (201). There are multiple sites of PadA binding to GPIIb/IIIa, resulting in platelet adhesion, dense granule secretion, and spreading on immobilized *S. gordonii* (201). However, PadA is dispensable for *S. gordonii* -platelet aggregation but is essential for adhesion of bacteria to platelets (202).

PadA and Hsa are needed for *S. gordonii* binding to cellular fibronectin and vitronectin, and to promote the formation of biofilms (203). Platelets can bind to immobilized *S. gordonii* through GPIIb/IIIa and GPIbα through PadA and Hsa, respectively (204). *S. gordonii* expresses two cell wall-associated polypeptides, streptococcal surface protein A and B (SspA and SspB, belonging to the antigen 1/antigen 2 family) (205). Both of these proteins induce GPIIb/IIIa-dependent aggregation and their deletion extends the lag time for platelet aggregation but does not affect adhesion to platelets (61, 205).

*S. gordonii-*mediated platelet aggregation also involves FcγRllA (206). The activation of FcγRIIA is dependent on IgG binding and GPIIb/IIIa involvement (77). Platelet releasate is essential, with released ADP and thromboxane A_2_ being needed for platelet aggregation by *S. gordonii* (77). Conversely, released PF4 binds to bacteria and reduces the lag time for platelet aggregation by *S. gordonii* (77). **Figure 2D** summarizes the main *S. gordonii* proteins that can activate platelets.

## Platelet interactions with *Porphyromonas gingivalis*

*P. gingivalis* is a gram-negative, anaerobic bacterium that is the major cause of periodontitis (207). *P. gingivalis* infection can increase thrombosis risks in patients with atherosclerosis, ischemic stroke, aneurysm, and atrial fibrillation (208–211). *P. gingivalis* has multiple effects on platelets. Platelets can endocytose *P. gingivalis* without the need for other agonists (*i.e.*, ADP) (212). *P. gingivalis* secretes cysteine proteinases called gingipains, which have trypsin-like activity (213) and are essential for *P. gingivalis* virulence (214). Through gingipains, *P. gingivalis* enhances pneumococcal adhesion to alveoli by inducing PAFR expression (133). There are two types of gingipains; lysine-specific protease (Kgp) and three variants of the arginine-specific protease (Rgp): RgpA_cat_, RgpB, and high-molecular-weight arginine-specific gingipain A (HRgpA) (215). RgpB and HRgpA induce platelet activation and aggregation by activating PAR1 and PAR4 (213). The incubation of *P. gingivalis* with human whole blood increased the potential of thrombosis (216). *P. gingivalis* induces platelet aggregation, P-selectin expression, platelet neutrophil aggregation, and NET formation (217). This effect can or cannot be modified by the addition of ADP (217). In addition, *P. gingivalis* increases the free calcium concentration in platelets and induces the release of RANTES from platelets, but at the same time, it can degrade it (218). Rgp and Kgp express a gingipain-derived hemagglutinin domain (Hgp44) at the C-termini, which undergoes autoproteolytic cleavage (219). Hgp44 was shown to be essential for platelet aggregation and activation (219). Also, LPS isolated from *P. gingivalis* enhances platelet spreading and filopodial extensions (220). The increase of filopodial extensions is mediated by the activation of Cdc42, which is a small GTPase that is essential for filopodial formation (220). Thus, RgpB, HRgpA, Hgp44, and LPS, produced by *P. gingivalis* can induce platelet aggregation and activation. **Figure 2E** summarizes the main *P. gingivalis* proteins that can activate platelets.

## Platelet interactions with *Helicobacter pylori*

*H. pylori* is a gram-negative bacterium known for its role in peptic ulcers, but it also contributes to cardiovascular diseases (CVDs; *e.g.*, myocardial infarction (MI), atherosclerosis, and immune thrombocytopenic purpura (ITP)) (221). *H. pylori*-infected patients develop chronic ITP, which is a result of platelet destruction by autoantibodies (221). *H. pylori* can cause thrombocytopenia without preceding bacteremia through a mechanism mediated by autoantibodies that destroy platelets (5). Consistently, patients treated with *H. pylori* eradication therapy have increased platelet counts (222). The development of thrombocytopenia involves the *H. pylori* low-molecular-weight antigen (Lpp20), which binds to platelets and can specifically react with sera from patients with *H. pylori* to induce chronic ITP (**Figure 2F**) (223). *H. pylori* requires the presence of plasma proteins, such as vWF and specific IgGs, to induce platelet aggregation and activation (61, 224). Function-blocking antibodies against vWF or GPIba inhibited *H. pylori*-induced platelet aggregation (224). This was confirmed in Bernard Soulier Syndrome patients who failed to respond to *H. pylori* (61). However, as with any bacteria, some strains of *H. pylori* activate platelets, and some do not (221).

## Platelet interactions with *Staphylococcus epidermidis*

*S. epidermidis*, is a coagulase-negative strain present on skin that can cause endocarditis and infections of medical-implemented devices (82). *S. epidermidis* can cause fibrin clot rupture, which leads to infected clot embolization and cause systemic infection (225). In general, coagulase-negative staphylococci are less virulent than positive bacteria such as *S. aureus* (226). *S. epidermidis* can, directly and indirectly, interact with platelets through the serine aspartate dipeptide repeat G (SdrG), which is an MSCRAMM (82). The direct interaction involves GPIIb/IIIa and indirect interaction involves fibrinogen, IgG, and FcγRII (82). *S. epidermidis* can crosslink GPIIb/IIIa and FcγRIIA to activate platelets (82). **Figure 2G** summarizes the main *P. gingivalis* proteins that can activate platelets.

## Bacterial stimulation of platelets and its clinical significance

While platelet activation plays a key role in helping the body eliminate viral and bacterial infections, excessive platelet stimulation can worsen disease outcomes, particularly in conditions like IE and sepsis. In preclinical studies for IE and sepsis, antiplatelet therapy such as aspirin has shown promising results when used as prophylactic or adjunct therapy (227–231). In both sepsis and IE, platelets are essential in the first line to remove pathogens. However, once the infection is established, platelet activation can worsen the condition. Extensive activation leads to thrombotic events that exacerbate the infection, promote bacterial survival, and ultimately harm the patient. Therefore, in theory, inhibiting platelet activity should be beneficial. As a result, several prospective and retrospective clinical studies have investigated whether antiplatelet therapies can reduce infection-related complications. However, no clear conclusions have yet been reached regarding their effectiveness in preventing or slowing the progression of infection (232–241). The main limitations of these studies include small sample sizes, which make it challenging to achieve statistical significance, as well as significant variability in patient age, underlying health conditions, the duration and dosage of antiplatelet therapy before or after the onset of infection, and the bacterial strains responsible for the disease (242). Despite the complexity of platelet–bacteria interactions, current research provides a solid foundation for future clinical applications. For example, platelet activation markers could be used as early diagnostic or prognostic tools in sepsis or IE, while targeted modulation of platelet responses could help reduce pathological thrombosis without compromising immune defense. An ideal target would be FcγRIIA receptor on the platelet surface since it is needed for pathogen-induced platelet activation (53). Furthermore, understanding specific bacterial virulence factors that alter platelet function opens new opportunities for precision medicine, where therapies are tailored based on the infecting pathogen’s profile. Ultimately, integrating platelet-related findings into clinical practice holds promise for improving the management and outcomes of severe bacterial infections.

## Conclusion

**Figure 3:**
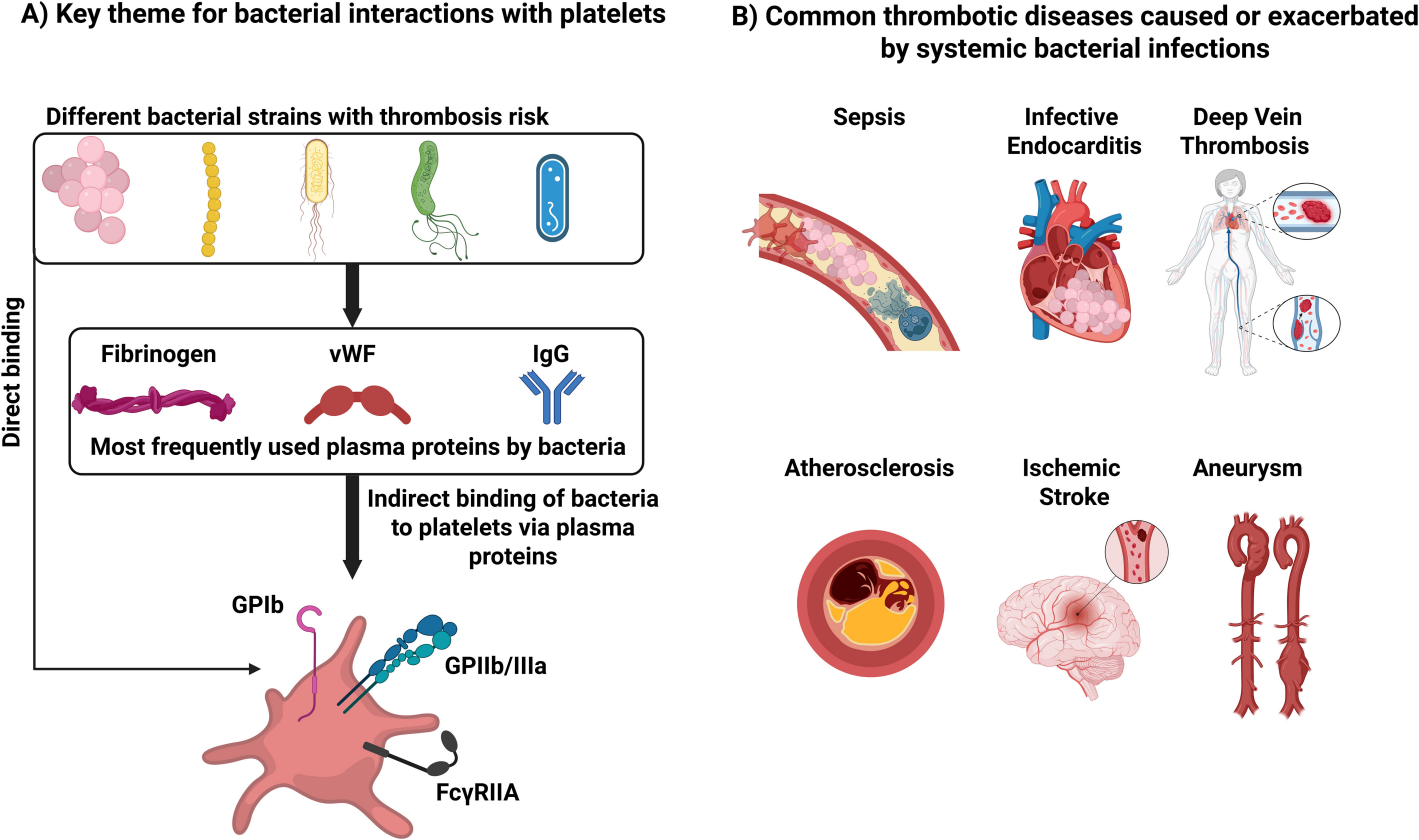
Key themes for bacterial interactions with platelets and cardiovascular disease. **(A)** Interactions that can lead to thrombosis. **(B)** Common thrombotic diseases caused or exacerbated by systemic bacterial infections.

Our understanding of platelet functions as immune cells has dramatically expanded, suggesting that they are crucial to responses to microbial infections. The interactions between platelets and pathogens are dynamic, multifaceted, and complicated processes that involve host defense mechanisms and microbial evasion strategies. Bacteria have also evolved mechanisms to exploit platelet functions for their benefit. While some bacteria have surface molecules that facilitate their adhesion and activation of platelets, others do not. Instead, some bacteria use plasma proteins as adapters to connect them with platelets. The most frequently exploited plasma proteins are fibrinogen, IgG, and vWF. These bind to GPIb and GPIIb/IIIa, which are frequently involved in the direct interaction between platelets and bacteria (**Figure 3**). However, these interactions alone are generally not sufficient to trigger platelet activation. For most bacterial species, platelet activation relies on FcγRIIa signaling. Inhibiting FcγRIIa—either through antibody blockade or depletion of specific IgG—effectively prevents platelet activation. This demonstrates that the interaction between IgG and FcγRIIa is crucial for initiating platelet activation. This is a unique feature of the immune response of platelets. For hemostasis, one type of receptor activation is sufficient to activate platelets.

Throughout this review, we have highlighted how most of these interactions lead to platelet consumption, dysregulated immune responses, or exacerbation of thrombotic events (**Table 1**). However, platelets are also involved in immune responses against bacteria and in their eradication. The presence of platelets is essential for TNF- α release following LPS injection in mice. Platelets are important in preventing liver injury during *S. aureus* infection. Platelets are key to neutrophil activation and preventing *S. pneumoniae* propagation. Platelets are capable of endocytosing and killing certain bacteria such as *S. aureus* and *E. coli*. Despite these insights, the importance of the platelet immune response against bacterial infection is still understudied. A major challenge in the field is the absence of FcγRIIa on mouse platelets. In mice, bacteria-driven platelet activation does not rely on FcγRIIa and is likely to follow mechanisms that might be distinct from humans. Other challenges also include contradictory findings, perhaps more reflective of the assays used or other technical issues such as the form of platelets used (washed platelets or PRP), platelets to bacteria ratio, bacterial strains, incubation times and temperature, incubation condition (static or stirring), platelet isolation methods, and platelet activation assay read out (aggregation or P-selectin exposure). It is hoped that our summary of the strategies bacteria use to affect platelets will help guide the needed research into the mechanisms underlying these effects.

**Table 1:** Summary of the bacterial proteins that interact with platelets either through direct interactions, released toxins, or via bridging plasma proteins.

Bacteria	Bacterial protein	Binding protein	Platelet receptor/s or proteins
*Staphylococcus aureus*	Direct Interaction		
	Iron-responsive surface determinant B (IsdB)	–	GPIIb/IIIa
	Staphylococcal accessory regulator protein (SarA)	–	GPIb
	Serine-rich adhesin protein (SraP)	–	GPIb?
	Serine-aspartate repeat protein (SdrE)	–	–
	Lipoteichoic acid	–	PAFR
	Staphylococcal protein A (SpA)	–	gC1qR-p33
	Indirect Interactions (via plasma proteins)		
	Staphylococcal protein A (SpA)	vWF	GPIb
		Fc region of IgG	FcγRllA
	Clumping factors A and B (ClfA and ClfB)	fibrinogen	GPIIb/IIIa
		IgG	FcγRIIA
		Complement proteins	Unknown receptor
	Fibronectin-binding proteins A and B (FnBPA and FnBPB)	Fibrinogen	GPIIb/IIIa
		IgG	FcγRIIA
	Extracellular fibrinogen binding protein (Efb)	C3	Unknown receptor
		Fibrinogen	GPIIb/IIIa
		–	P-Selectin
	vWF-Binding Protein (vWbp)	vWF	Unknown receptor
	Staphylokinase	Plasminogen	–
	Indirect Interactions (via secreted proteins)		
	α-toxin	–	ADAM10
	Toxic shock syndrome toxin-1 (TSST-1)	–	–
	Panton-valentine leucocidin (PVL)	–	–
	Extracellular adherence protein (Eap)	–	Platelet-surface thiol isomerases, e.g., PDI, ERp57, and ERp72
	Chemotaxis inhibitory protein of S. aureus (CHIPS)	–	–
	Formyl peptide receptor-like 1 inhibitory protein (FLIPr)	–	–
	Staphylococcal complement inhibitor (SCIN)	–	–
	Major autolysin (AtlA)	–	–
	Superantigen-like-5 (SSL-5)	–	GPIb, GPIIb/IIIa and GPVI
	Staphylocoagulase	–	–
*Streptococcus pneumoniae*	Pneumolysin (Ply)	–	–
	Phosphorylcholine	–	Platelet-activating factor receptor (PAFR)?
	Peptidoglycan and pneumoniae endopeptidase O (PepO)	–	TLR2 and TLR4
	Neuraminidases A, B, and C (NanA, NanB, and NanC)	–	Sialic acid
	Pneumococcal adherence and virulence factor B (PavB) and pneumococcal surface protein C (PspC)	Thrombospondin-1 (TSP-1)	GPIIb/IIIa
*Escherichia coli*	Lipopolysaccharide (LPS)	–	TLR4
	Shiga toxin	–	(Globotriaosylceramide 3 and 4 receptors (Gb3 and Gb4))
*Streptococcus sanguinis*	Platelet-associated aggregation protein (PAAP)	–	GPIIb/IIIa, GPIb or uncharacterized receptor??
	Serine-rich protein A (SrpA) and hemagglutinin salivary antigen (Hsa)	–	GPIb
*Streptococcus gordonii*	Gordonii surface proteins glycosylated streptococcal protein B (GspB)	–	GPIb
	Hemagglutinin salivary adhesin (Hsa)	–	GPIb and GPIIb/IIIa
	Serine-rich protein A (SrpA)	–	GPIb and GPIIb/IIIa
	Platelet adherence protein A (PadA)	–	GPIIb/IIIa
	Streptococcal surface protein A and B (SspA and SspB)	–	GPIIb/IIIa
*Porphyromonas gingivalis*	Arginine-specific protease B (RgpB) and high-molecular-weight arginine-specific gingipain A (HRgpA)	–	PAR1 and PAR4
	Hemagglutinin/adhesion domain of the Arg-gingipain A protein (Hgp44)	–	–
	LPS	–	TLR4
*Helicobacter pylori*	Low molecular weight antigen (Lpp20)	–	–
*Staphylococcus epidermidis*	Serine aspartate dipeptide repeat G (SdrG)	–	GPIIb/IIIa
		Fibrinogen	GPIIb/IIIa
		IgG	FcγRIIA
